# 7-Hy­droxy-6-meth­oxy-2*H*-chromen-2-one

**DOI:** 10.1107/S1600536810029296

**Published:** 2010-07-31

**Authors:** Hooi-Kheng Beh, Zhari Ismail, Mohd Zaini Asmawi, Wan-Sin Loh, Hoong-Kun Fun

**Affiliations:** a School of Pharmaceutical Sciences, Universiti Sains Malaysia, 11800 USM, Penang, Malaysia; b X-ray Crystallography Unit, School of Physics, Universiti Sains Malaysia, 11800 USM, Penang, Malaysia

## Abstract

The title compound, C_10_H_8_O_4_, is one of the coumarins existing in *Morinda citrifolia L* (Noni). The chromenone ring system is approximately planar with a maximum deviation of 0.0208 (14) Å. The meth­oxy group does not deviate from this plane [C—O—C—C torsion angle = −1.5 (3)°], indicating that the whole mol­ecule is almost planar. In the crystal packing, inter­molecular O—H⋯O hydrogen bonds link the mol­ecules into chains. These are further connected by C—H⋯O hydrogen bonds.

## Related literature

For background and the biological activity of *Morinda citrifolia L*, see: Wang *et al.* (2002 ▶); Samoylenko *et al.* (2006 ▶); Silva *et al.* (2001 ▶); Goy *et al.* (1993 ▶); Cassady *et al.* (1979 ▶); Shaw *et al.* (2003 ▶); Ding *et al.* (2008 ▶). For the stability of the temperature controller used in the data collection, see: Cosier & Glazer (1986 ▶).
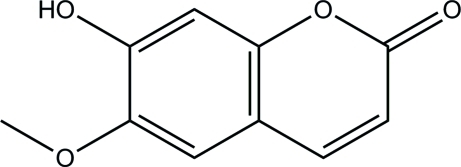


## Experimental

### 

#### Crystal data

C_10_H_8_O_4_
*M* *_r_* = 192.16
Orthorhombic, 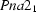

*a* = 7.0771 (2) Å
*b* = 17.3485 (4) Å
*c* = 6.9672 (2) Å
*V* = 855.41 (4) Å^3^
*Z* = 4
Mo *K*α radiation
μ = 0.12 mm^−1^
*T* = 100 K
0.39 × 0.11 × 0.08 mm

#### Data collection

Bruker SMART APEXII CCD area-detector diffractometer
Absorption correction: multi-scan (*SADABS*; Bruker, 2009 ▶) *T*_min_ = 0.956, *T*_max_ = 0.991
9630 measured reflections
1364 independent reflections
1213 reflections with *I* > 2σ(*I*)
*R*_int_ = 0.035

#### Refinement

*R*[*F* ^2^ > 2σ(*F* ^2^)] = 0.037
*wR*(*F* ^2^) = 0.094
*S* = 1.07
1364 reflections
132 parameters
1 restraint
H atoms treated by a mixture of independent and constrained refinement
Δρ_max_ = 0.33 e Å^−3^
Δρ_min_ = −0.26 e Å^−3^

### 

Data collection: *APEX2* (Bruker, 2009 ▶); cell refinement: *SAINT* (Bruker, 2009 ▶); data reduction: *SAINT*; program(s) used to solve structure: *SHELXTL* (Sheldrick, 2008 ▶); program(s) used to refine structure: *SHELXTL*; molecular graphics: *SHELXTL*; software used to prepare material for publication: *SHELXTL* and *PLATON* (Spek, 2009 ▶).

## Supplementary Material

Crystal structure: contains datablocks global, I. DOI: 10.1107/S1600536810029296/bt5305sup1.cif

Structure factors: contains datablocks I. DOI: 10.1107/S1600536810029296/bt5305Isup2.hkl

Additional supplementary materials:  crystallographic information; 3D view; checkCIF report

## Figures and Tables

**Table 1:** Hydrogen-bond geometry (Å, °)

*D*—H⋯*A*	*D*—H	H⋯*A*	*D*⋯*A*	*D*—H⋯*A*
O3—H1*O*3⋯O2^i^	0.92 (3)	1.85 (3)	2.6558 (17)	146 (3)
C5—H5*A*⋯O2^ii^	0.93	2.48	3.345 (2)	154

Symmetry codes: (i) 
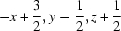
; (ii) 
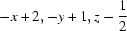
.

**Fig. 1.**
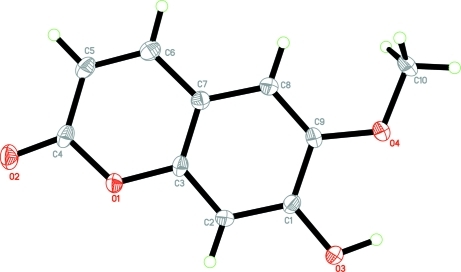
The molecular structure of the title compound, showing 50% probability displacement ellipsoids and the atom-numbering scheme.

**Fig. 2.**
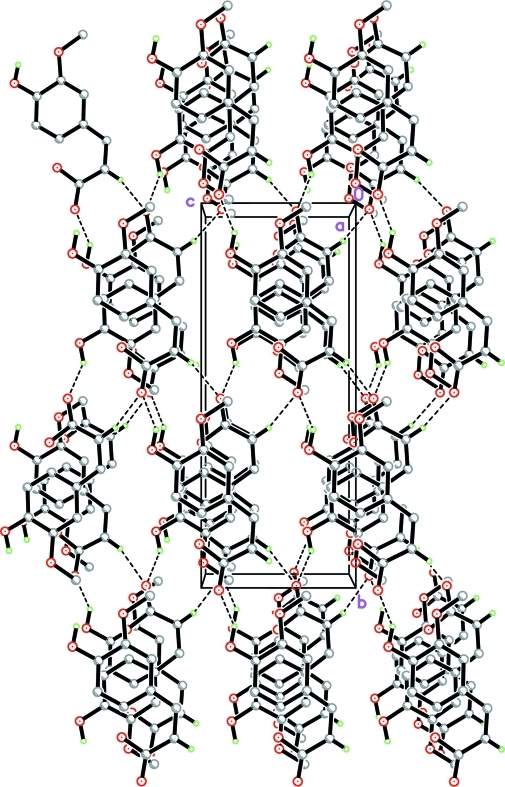
The crystal packing of the title compound, viewed along the a axis, showing the two-dimensional planes. Intermolecular interactions are shown as dashed lines. H atoms not involved in the intermolecular interactions (dashed lines) have been omitted for clarity.

## supplementary crystallographic information

### Comment

*Morinda citrifolia L* (Noni) has been used in folk remedies by Polynesians for over 2000 years (Wang *et al.*, 2002). 7-Hydroxy-6-methoxy-2*H*-chromen-2-one (Scopoletin), a yellow to beige crystalline powder, is one of the coumarins present in *Morinda citrifolia*. The reference (Samoylenko *et al.*, 2006) suggested Scopoletin as a marker constituent for quality control of Noni. This compound is reported to have a broad range of therapeutic effects including antimicrobial (Silva *et al.*, 2001; Goy *et al.*, 1993), antitumor (Cassady *et al.*, 1979), antioxidant (Shaw *et al.*, 2003), anti-inflammatory (Ding *et al.*, 2008) properties.

In the title compound, Fig. 1, the chromenone ring system (C1–C9/O1/O2) is approximately planar with a maximum deviation of 0.0208 (14) Å at atom C5. This mean plane forms a dihedral angle of 1.67 (8)° with the methoxy group (O4/C10) attached to it, indicating that the whole molecule is almost planar.

In the crystal packing, Fig. 2, intermolecular O3—H1O3···O2 and C5—H5A···O2 hydrogen bonds (Table 1) link the molecules into two-dimensional planes parallel to *bc* plane.

### Experimental

The raw materials of *Morinda citrifolia* were collected from Kampung Seronok, Penang, Malaysia. A voucher specimen (No. 10612) has been deposited at the herbarium of the School of Biological Sciences, Universiti Sains Malaysia. The plant samples were cleaned with water and dried in oven at 55°C for 3 days. The dried powdered fruit of *Morinda citrifolia* was repeatedly extracted by soxhlet extractor by using fresh methanol for 5 days. The pooled methanol extracts were evaporated to yield 18.0% residue. A portion of these methanolic extracts was reconstituted in distilled water and partitioned sequentially with equal volume of chloroform (CHCl_3_), ethyl acetate (EA) and n-butanol (BuOH). The eluates were dried to yield 11.1%, 9.0%, 20.2% of CHCl_3_ fraction, EA fraction and BuOH fraction respectively. The CHCl_3_ fraction was subjected to column chromatography and was eluted sequentially with of petroleum ether, petroleum ether-chloroform mixtures (99:1, 95:5, 90:10, 85:15; 80:20, 75:25, 70:30, 65:35, 60:40, 55:45, 50:50, 40:60, 30:70, 20:80, 10:90), chloroform and chloroform-methanol mixtures (99:1, 95:5, 90:10, 85:15; 80:20, 75:25, 70:30, 65:35, 60:40, 55:45, 50:50, 40:60, 30:70, 20:80, 10:90) and methanol. Fractions eluted from the petroleum ether-chloroform mixture (90:10) yielded a yellowish-orange amorphous powder (82.5 mg). Yellow colour crystals suitable for X-ray crystallography were obtained upon repeated recrystallization with chloroform. The molecular weight of the titled compound found to be 192 and the melting point is 477–479 K.

### Refinement

The H atom bonded to O was located from a difference Fourier map and was refined freely [O–H = 0.92 (3) Å]. The remaining H atoms were positioned geometrically [C–H = 0.93 or 0.96 Å] and were refined using a riding model, with *U*_iso_(H) = 1.2 or 1.5 *U*_eq_(C). A rotating group model was applied to the methyl group. In the absence of significant anomalous dispersion, 879 Friedel pairs were merged for the final refinement.

### Crystal data

**Table:**

C_10_H_8_O_4_	*F*(000) = 400
*M**_r_* = 192.16	*D*_x_ = 1.492 Mg m^−^^3^
Orthorhombic, *P**n**a*2_1_	Mo *K*α radiation, λ = 0.71073 Å
Hall symbol: P 2c -2n	Cell parameters from 3125 reflections
*a* = 7.0771 (2) Å	θ = 2.4–29.9°
*b* = 17.3485 (4) Å	µ = 0.12 mm^−^^1^
*c* = 6.9672 (2) Å	*T* = 100 K
*V* = 855.41 (4) Å^3^	Needle, yellow
*Z* = 4	0.39 × 0.11 × 0.08 mm

### Data collection

**Table:**

Bruker SMART APEXII CCD area-detector diffractometer	1364 independent reflections
Radiation source: fine-focus sealed tube	1213 reflections with *I* > 2σ(*I*)
graphite	*R*_int_ = 0.035
φ and ω scans	θ_max_ = 30.3°, θ_min_ = 2.4°
Absorption correction: multi-scan (*SADABS*; Bruker, 2009)	*h* = −10→9
*T*_min_ = 0.956, *T*_max_ = 0.991	*k* = −24→24
9630 measured reflections	*l* = −9→8

### Refinement

**Table:**

Refinement on *F*^2^	Primary atom site location: structure-invariant direct methods
Least-squares matrix: full	Secondary atom site location: difference Fourier map
*R*[*F*^2^ > 2σ(*F*^2^)] = 0.037	Hydrogen site location: inferred from neighbouring sites
*wR*(*F*^2^) = 0.094	H atoms treated by a mixture of independent and constrained refinement
*S* = 1.07	*w* = 1/[σ^2^(*F*_o_^2^) + (0.0496*P*)^2^ + 0.1813*P*] where *P* = (*F*_o_^2^ + 2*F*_c_^2^)/3
1364 reflections	(Δ/σ)_max_ = 0.001
132 parameters	Δρ_max_ = 0.33 e Å^−^^3^
1 restraint	Δρ_min_ = −0.26 e Å^−^^3^

### Special details

**Table:**

Experimental. The crystal was placed in the cold stream of an Oxford Cryosystems Cobra open-flow nitrogen cryostat (Cosier & Glazer, 1986) operating at 100.0 (1) K.
Geometry. All e.s.d.'s (except the e.s.d. in the dihedral angle between two l.s. planes) are estimated using the full covariance matrix. The cell e.s.d.'s are taken into account individually in the estimation of e.s.d.'s in distances, angles and torsion angles; correlations between e.s.d.'s in cell parameters are only used when they are defined by crystal symmetry. An approximate (isotropic) treatment of cell e.s.d.'s is used for estimating e.s.d.'s involving l.s. planes.
Refinement. Refinement of *F*^2^ against ALL reflections. The weighted *R*-factor *wR* and goodness of fit *S* are based on *F*^2^, conventional *R*-factors *R* are based on *F*, with *F* set to zero for negative *F*^2^. The threshold expression of *F*^2^ > σ(*F*^2^) is used only for calculating *R*-factors(gt) *etc*. and is not relevant to the choice of reflections for refinement. *R*-factors based on *F*^2^ are statistically about twice as large as those based on *F*, and *R*- factors based on ALL data will be even larger.

### Fractional atomic coordinates and isotropic or equivalent isotropic displacement parameters (Å^2^)

**Table:**

	*x*	*y*	*z*	*U*_iso_*/*U*_eq_	
O1	0.82231 (19)	0.37644 (6)	0.5023 (2)	0.0161 (3)	
O2	0.8589 (2)	0.49194 (7)	0.3781 (2)	0.0221 (3)	
O3	0.7202 (2)	0.13880 (7)	0.8064 (2)	0.0223 (3)	
O4	0.81670 (19)	0.05917 (7)	0.4922 (2)	0.0199 (3)	
C1	0.7724 (3)	0.17732 (9)	0.6463 (3)	0.0153 (3)	
C2	0.7725 (3)	0.25710 (9)	0.6524 (3)	0.0147 (3)	
H2A	0.7382	0.2832	0.7636	0.018*	
C3	0.8251 (2)	0.29720 (9)	0.4884 (3)	0.0144 (3)	
C4	0.8665 (2)	0.42295 (9)	0.3488 (3)	0.0171 (4)	
C5	0.9176 (3)	0.38545 (10)	0.1709 (3)	0.0182 (4)	
H5A	0.9461	0.4152	0.0637	0.022*	
C6	0.9243 (2)	0.30757 (10)	0.1584 (3)	0.0171 (3)	
H6A	0.9606	0.2846	0.0437	0.021*	
C7	0.8762 (3)	0.26016 (9)	0.3195 (3)	0.0145 (3)	
C8	0.8758 (2)	0.17879 (9)	0.3153 (3)	0.0151 (3)	
H8A	0.9099	0.1529	0.2037	0.018*	
C9	0.8248 (3)	0.13773 (9)	0.4770 (3)	0.0148 (3)	
C10	0.8609 (3)	0.01496 (9)	0.3244 (3)	0.0218 (4)	
H10A	0.8482	−0.0389	0.3527	0.033*	
H10B	0.9884	0.0256	0.2854	0.033*	
H10C	0.7758	0.0286	0.2226	0.033*	
H1O3	0.712 (4)	0.0869 (16)	0.782 (5)	0.048 (8)*	

### Atomic displacement parameters (Å^2^)

**Table:**

	*U*^11^	*U*^22^	*U*^33^	*U*^12^	*U*^13^	*U*^23^
O1	0.0216 (6)	0.0117 (5)	0.0150 (6)	−0.0002 (4)	−0.0009 (6)	0.0010 (5)
O2	0.0300 (7)	0.0133 (5)	0.0230 (8)	−0.0011 (5)	−0.0046 (6)	0.0033 (5)
O3	0.0378 (8)	0.0135 (5)	0.0157 (7)	−0.0024 (5)	0.0077 (7)	0.0012 (6)
O4	0.0300 (7)	0.0116 (5)	0.0182 (7)	−0.0008 (5)	0.0038 (6)	−0.0021 (6)
C1	0.0174 (8)	0.0150 (7)	0.0135 (9)	−0.0022 (6)	−0.0016 (8)	0.0022 (7)
C2	0.0175 (8)	0.0149 (7)	0.0117 (8)	−0.0005 (6)	0.0008 (8)	−0.0013 (7)
C3	0.0153 (7)	0.0117 (6)	0.0162 (9)	−0.0009 (6)	−0.0027 (7)	0.0029 (8)
C4	0.0170 (8)	0.0158 (7)	0.0185 (10)	−0.0023 (6)	−0.0039 (7)	0.0064 (7)
C5	0.0196 (8)	0.0193 (7)	0.0156 (9)	−0.0019 (6)	−0.0017 (8)	0.0056 (7)
C6	0.0166 (8)	0.0199 (7)	0.0149 (8)	−0.0002 (6)	−0.0007 (8)	0.0022 (7)
C7	0.0147 (8)	0.0150 (7)	0.0138 (9)	0.0003 (6)	−0.0015 (8)	0.0006 (7)
C8	0.0170 (8)	0.0150 (7)	0.0134 (8)	−0.0005 (6)	0.0006 (8)	−0.0010 (7)
C9	0.0158 (8)	0.0112 (6)	0.0173 (9)	−0.0001 (6)	−0.0010 (7)	−0.0008 (7)
C10	0.0284 (9)	0.0148 (7)	0.0223 (10)	0.0015 (6)	0.0034 (9)	−0.0064 (8)

### Geometric parameters (Å, °)

**Table:**

O1—C4	1.376 (2)	C4—C5	1.446 (3)
O1—C3	1.3781 (18)	C5—C6	1.355 (2)
O2—C4	1.215 (2)	C5—H5A	0.9300
O3—C1	1.352 (2)	C6—C7	1.432 (2)
O3—H1O3	0.92 (3)	C6—H6A	0.9300
O4—C9	1.3682 (18)	C7—C8	1.412 (2)
O4—C10	1.433 (2)	C8—C9	1.381 (3)
C1—C2	1.385 (2)	C8—H8A	0.9300
C1—C9	1.415 (3)	C10—H10A	0.9600
C2—C3	1.388 (3)	C10—H10B	0.9600
C2—H2A	0.9300	C10—H10C	0.9600
C3—C7	1.389 (3)		
			
C4—O1—C3	121.82 (16)	C5—C6—C7	120.94 (18)
C1—O3—H1O3	111 (2)	C5—C6—H6A	119.5
C9—O4—C10	117.42 (16)	C7—C6—H6A	119.5
O3—C1—C2	117.99 (16)	C3—C7—C8	118.68 (16)
O3—C1—C9	121.33 (15)	C3—C7—C6	117.39 (14)
C2—C1—C9	120.68 (16)	C8—C7—C6	123.93 (17)
C1—C2—C3	118.44 (16)	C9—C8—C7	119.95 (16)
C1—C2—H2A	120.8	C9—C8—H8A	120.0
C3—C2—H2A	120.8	C7—C8—H8A	120.0
O1—C3—C2	116.00 (17)	O4—C9—C8	126.04 (17)
O1—C3—C7	121.65 (16)	O4—C9—C1	114.05 (17)
C2—C3—C7	122.35 (14)	C8—C9—C1	119.90 (14)
O2—C4—O1	115.91 (18)	O4—C10—H10A	109.5
O2—C4—C5	126.75 (17)	O4—C10—H10B	109.5
O1—C4—C5	117.34 (14)	H10A—C10—H10B	109.5
C6—C5—C4	120.84 (17)	O4—C10—H10C	109.5
C6—C5—H5A	119.6	H10A—C10—H10C	109.5
C4—C5—H5A	119.6	H10B—C10—H10C	109.5
			
O3—C1—C2—C3	−179.72 (16)	C2—C3—C7—C6	178.81 (16)
C9—C1—C2—C3	0.1 (3)	C5—C6—C7—C3	−0.8 (3)
C4—O1—C3—C2	−178.29 (15)	C5—C6—C7—C8	178.45 (16)
C4—O1—C3—C7	1.2 (2)	C3—C7—C8—C9	0.2 (2)
C1—C2—C3—O1	179.84 (14)	C6—C7—C8—C9	−179.06 (17)
C1—C2—C3—C7	0.3 (3)	C10—O4—C9—C8	−1.5 (3)
C3—O1—C4—O2	179.90 (15)	C10—O4—C9—C1	177.71 (15)
C3—O1—C4—C5	−0.3 (2)	C7—C8—C9—O4	179.38 (15)
O2—C4—C5—C6	178.64 (18)	C7—C8—C9—C1	0.2 (3)
O1—C4—C5—C6	−1.1 (3)	O3—C1—C9—O4	0.2 (3)
C4—C5—C6—C7	1.7 (3)	C2—C1—C9—O4	−179.60 (15)
O1—C3—C7—C8	−179.93 (14)	O3—C1—C9—C8	179.42 (15)
C2—C3—C7—C8	−0.5 (3)	C2—C1—C9—C8	−0.4 (3)
O1—C3—C7—C6	−0.7 (2)		

### Hydrogen-bond geometry (Å, °)

**Table:**

*D*—H···*A*	*D*—H	H···*A*	*D*···*A*	*D*—H···*A*
O3—H1O3···O2^i^	0.92 (3)	1.85 (3)	2.6558 (17)	146 (3)
C5—H5A···O2^ii^	0.93	2.48	3.345 (2)	154

Symmetry codes: (i) −*x*+3/2, *y*−1/2, *z*+1/2; (ii) −*x*+2, −*y*+1, *z*−1/2.
